# Influenza vaccination attenuates acute myocardial infarction and stroke risk following influenza infection: a register-based, self-controlled case series study, Denmark, 2014 to 2025

**DOI:** 10.2807/1560-7917.ES.2026.31.13.2500706

**Published:** 2026-04-02

**Authors:** Roberto Croci, Johanna J Young, Hanne-Dorthe Emborg, Palle Valentiner-Branth, Steen Ethelberg, Christian Holm Hansen

**Affiliations:** 1 ECDC Fellowship Programme, Field Epidemiology path (EPIET), European Centre for Disease Prevention and Control (ECDC), Stockholm, Sweden; 2 Department of Infectious Disease Epidemiology and Prevention, Statens Serum Institut, Copenhagen, Denmark; 3 Department of Public Health, Global Health Section, University of Copenhagen, Copenhagen, Denmark; * These authors contributed equally to this work and share first authorship.

**Keywords:** laboratory-confirmed influenza, myocardial infarction, influenza vaccination, stroke, self-controlled case series

## Abstract

**BACKGROUND:**

Influenza infection is a recognised trigger of acute myocardial infarction (AMI) and stroke, but whether influenza vaccination modifies this risk remains unclear.

**AIM:**

We aimed to quantify the short-term cardiovascular risk after laboratory-confirmed influenza infection and assess whether vaccination attenuates it.

**METHODS:**

We conducted a nationwide self-controlled case series study using Danish health registries (2014–2025) and included individuals aged ≥ 40 years with a first-ever hospital admission for AMI or stroke within ± 365 days of a PCR-confirmed influenza infection. We defined days 1–7 after specimen date as the risk period and excluded a 14-day pre-exposure period to reduce reverse causality, where influenza testing might be prompted by cardiovascular disease symptoms. We linked testing, hospitalisation, vaccination and mortality data deterministically via unique personal identifiers. We estimated incidence rate ratios (IRRs) and 95% confidence intervals (CI) with conditional Poisson regression.

**RESULTS:**

Among 1,221 individuals with a first-ever AMI (n = 429; 35%) or stroke (n = 792; 65%), median age was 75 years (interquartile range: 66–82); 561 (46%) were female. After calendar-month adjustment, the IRR for cardiovascular events during the risk period was 3.5 (95% CI: 2.6–4.7), higher for AMI (IRR = 4.7; 95% CI: 3.1–7.4) than stroke (IRR = 2.9; 95% CI: 2.0–4.2). Prior influenza vaccination during the same influenza season, recorded in 610 (50%) episodes, reduced the excess risk of AMI or stroke associated with influenza infection (interaction p = 0.020).

**CONCLUSIONS:**

Influenza infection conferred a transiently increased risk of first-time AMI and stroke. Vaccination substantially attenuated this risk, supporting its role in preventing cardiovascular complications after breakthrough infection.

**Key public health message**

**What did you want to address in this study and why?**

Catching influenza increases the short-term risk of heart attack and stroke. Influenza vaccination has been shown to reduce this risk by preventing infection, but it is unclear whether it also offers protection among people who become infected despite vaccination. We wanted to quantify how much more at risk of heart attack and stroke adults in Denmark are shortly after catching influenza, and to assess if prior vaccination can attenuate this risk.

**What have we learnt from this study?**

In adults 40 years or older in Denmark, hospital admissions for heart attack and stroke were more frequent in the first week after testing positive for influenza than during any other period in the year before and after their test, almost threefold for stroke and fivefold for heart attack. This increased risk was about half as high among people who tested positive for influenza but had received the influenza vaccine that season.

**What are the implications of your findings for public health?**

Influenza vaccination may offer cardiovascular protection even in instances when it does not prevent infection. If confirmed by additional studies in other settings, this would strengthen the case for prioritising influenza vaccination among people at risk of heart disease or stroke and would support refining recommendations across Europe.

## Introduction

Cardiovascular diseases pose a substantial clinical and economic burden. Globally, ischaemic heart disease and ischaemic stroke alone account for roughly 2,275 and 820 disability-adjusted life-years per 100,000 population, respectively [1]. In the European Union, cardiovascular diseases led to 22 hospital admissions per 1,000 population and an estimated total healthcare cost exceeding EUR 282 billion in 2021 [2].

Influenza infection can trigger acute cardiovascular events through short-lived systemic inflammation that favours a pro-thrombotic state and destabilises vulnerable atherosclerotic plaques [3]. Self-controlled case series studies, which compare event rates within individuals during prespecified risk time windows against their own baseline time [4], have consistently shown transient increases in cardiovascular risk after laboratory-confirmed influenza. A Canadian study reported a sixfold increase in acute myocardial infarction risk during the first 7 days after positive test results (incidence rate ratio (IRR) = 6.05; 95% confidence interval (CI): 3.86–9.50) [5]; estimates from Spain and the Netherlands are similar [6,7]. Studies employing finer temporal resolution have further characterised the risk profile, indicating that peak incidence increases within 3 days, then tapers back within 2–4 weeks [8,9].

Among mounting evidence suggesting that influenza vaccination reduces cardiovascular risk, a recent meta-analysis of randomised controlled trials estimated 32% lower risk [10]. Two successive self-controlled case-series studies in the United Kingdom demonstrated a 20–23% reduced incidence for both acute myocardial infarction and stroke [11,12]. In particular, the second study reported no evidence of sex-specific differences, and effects were slightly stronger among people vaccinated early in the influenza season [12]. A meta-analysis including these same two studies provided further evidence of the protective effect of vaccination (pooled IRR = 0.84 for acute myocardial infarction; 95% CI: 0.78–0.91) [13].

Current international guidelines recommend annual influenza vaccination for people with cardiovascular disease or major risk factors [14,15]. In Denmark, influenza vaccination is provided free of charge to all pregnant women in their second or third trimester, to all individuals 65 years or older, and to younger individuals with cardiovascular disease or other chronic conditions [16,17].

There is robust evidence that influenza vaccination reduces the risk of cardiovascular events by preventing influenza infection. However, to our knowledge, only one study has examined whether vaccination modifies cardiovascular risk after infection. The Canadian study by Kwong et al., which included 364 myocardial infarction hospitalisations, found no significant interaction with vaccination status when modelling the effect of influenza on cardiovascular risk (interaction p value = 0.85), possibly due to limited statistical power [5]. Whether vaccination attenuates the cardiovascular consequences of breakthrough infection, therefore, remains uncertain.

Addressing this knowledge gap is critical, as vaccination might confer a dual benefit: preventing infection and attenuating the resultant cardiovascular consequences when breakthrough infection occurs. Denmark’s national registries offer a unique opportunity to address this question. We conducted a nationwide self-controlled case series study aiming to quantify the excess risk of acute myocardial infarction and stroke immediately following laboratory-confirmed influenza, and to investigate whether prior influenza vaccination modifies this risk.

## Methods

### Study design, exposure and outcome ascertainment

In this self-controlled case-series study, we used routinely collected Danish register data.

**Figure 1:**
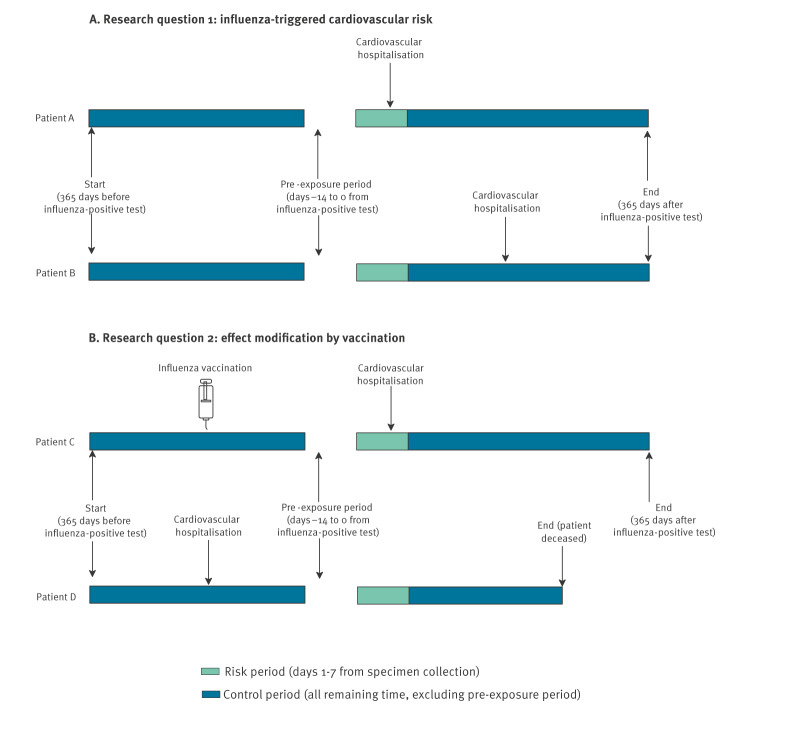
Overview of the self-controlled case series study design to assess the effect of influenza vaccination on acute myocardial infarction and stroke risk following influenza infection, Denmark, 2014–2025

We identified all laboratory-confirmed influenza infections that occurred during nine consecutive influenza seasons, that is, from 21 September 2015 to 3 March 2024. Around each infection, we applied a ± 365-day observation window to capture acute cardiovascular outcomes. As a result, the study period was 21 September 2014 to 3 March 2025. Figure 1 illustrates the study design.

Panel A: Patient A experiences an acute myocardial infarction or stroke during the risk period after the influenza-positive test date (day 0; risk interval shown in green). Patient B’s event occurs during the control period (blue). The study compares the within-person incidence during the risk period with that during the control period. Panel B: Patient C received a seasonal influenza vaccination more than 14 days before day 0, and their episode counts as vaccinated; Patient D is unvaccinated. The analysis contrasts post-infection cardiovascular risk between vaccinated and unvaccinated episodes. Patient D’s observation is censored at death before the prespecified end of the observation window. For both panels, the gaps denote the 14-day pre-exposure period (days −14 to 0, including day 0) during which no events are accrued. Observation windows are symmetric (−365 to +365 days around day 0) and are censored at death or study end. Diagrams not to scale.

### Exposure, outcome, and risk periods

We defined the specimen collection date for the positive influenza PCR test as the index date (day 0). To capture the acute effect of influenza on cardiovascular risk, we defined the primary risk period as days 1–7 post-index. To minimise reverse causality, we excluded a pre-exposure period spanning days −14 to 0, during which influenza testing may have been prompted by prodromal cardiovascular symptoms. The control period comprised all remaining observation time. Censoring occurred at death or study end, whichever came first.

### Study population

Eligible individuals were Danish residents aged 40 years or older who had at least one PCR-confirmed influenza infection during the relevant influenza seasons, and a first-ever hospital admission for acute myocardial infarction (International Classification of Diseases, 10th Revision (ICD-10) code I21) or stroke (I60, I61, I63) occurring within the episode’s symmetric observation window. Validation studies in Denmark have quantified the positive predictive values of these diagnostic codes at 97% (95% CI: 91–99) for first-time myocardial infarction and 69.3% (95% CI: 67.8–70.9) for stroke [18,19].

Each influenza infection constituted a separate episode with its own observation window, and overlap was permitted. However, since the study population was outcome-naïve by design, each individual could contribute only one first qualifying cardiovascular event to the analysis. If a cardiovascular event occurred in multiple observation windows, we selected the one with the closest index date or if tied, the earliest index date. We specified this rule a priori and applied it independent of vaccination status.

### Data linkage and data sources

We used the Central Person Register (CPR) number, a unique 10-digit identifier assigned to all Danish residents, to perform deterministic linkage across the following national health registries:

1. Danish Microbiology Database: influenza infections laboratory-confirmed by PCR, excluding repeat positives within 14 days of prior test and suspected false positives based on diagnostic metadata.
2. Danish National Patient Register: hospital admissions and diagnoses. To ensure only incident events, we excluded individuals with any prior acute myocardial infarction or stroke diagnosis before 21 September 2014.
3. Danish Vaccination Register: influenza vaccination dates.
4. Danish Mortality Monitoring System: death dates.

We derived information on age and gender from the CPR number. In Denmark, the last CPR digit encodes the registered gender, an even digit for female and odd for male. When individuals legally transition, a new CPR number is issued reflecting their affirmed gender [20]. As a consequence, our ‘gender’ variable captures ‘gender assigned at birth’ for cisgender individuals and ‘affirmed gender’ for transpersons who have legally transitioned. We report this operational definition in line with the Sex and Gender Equity in Research guidelines [21].

### Vaccination status

We defined an influenza season as running from International Organisation for Standardisation (ISO) week 21 of one calendar year through ISO week 20 of the next. For an episode to be considered vaccinated, the individual must have received the vaccine between ISO week 39 of one calendar year and ISO week 9 of the next within the same influenza season as the infection, and with the vaccination occurring at least 14 days before the index date. Otherwise, we considered the episode as unvaccinated.

### Statistical analysis

We fitted conditional Poisson regression models stratified by individual to estimate IRRs comparing event rates in risk and control periods. We set the logarithm of person-days as an offset to account for varying follow-up due to censoring by death. In the main analysis, we provide pooled IRRs as well as separate by disease, along with their 95% CI. We adjusted the models for calendar month as a categorical variable to control for seasonal effects in both influenza circulation and acute cardiovascular event occurrence. We also explored age adjustment approaches in dedicated analyses.

To test for effect modification by vaccination, we included an interaction term between the risk period indicator and vaccination status. This provided separate IRRs for vaccinated and unvaccinated episodes. We quantified effect modification via the ratio of these IRRs and assessed significance using likelihood-ratio tests comparing nested models with and without the interaction term. Full model specifications are provided in the Supplement.

We performed several additional analyses: (i) extended risk partitioning, including finer risk intervals (days 1–3, 4–7, 8–14, 15–28, 29–90 post-index); (ii) negative exposure control using *Campylobacter* spp. infection; (iii) negative outcome control using upper limb fracture (ICD-10 S42 and S52) and retinal detachment (ICD-10 H33) admissions; (iv) observation window shortening (± 180 and ± 90 days); (v) alternative control period definitions (pre-exposure or post-exposure only); (vi) excluding influenza detected during cardiovascular hospitalisation; (vii) shifting the index date backwards by 1, 2 or 3 days.

We prespecified the following subgroup analyses: excluding fatal events within 30 days of hospital admission, influenza A infections only, age 65 and younger only, separate effects in males and females, and atherosclerotic events only. We defined as ‘atherosclerotic’ all events excluding type 2 myocardial infarction and haemorrhagic stroke.

All statistical tests were two-sided, and we assessed significance at alpha = 0.05. We analysed data in R version 4.4.1.

## Results

### Study population

**Figure 2:**
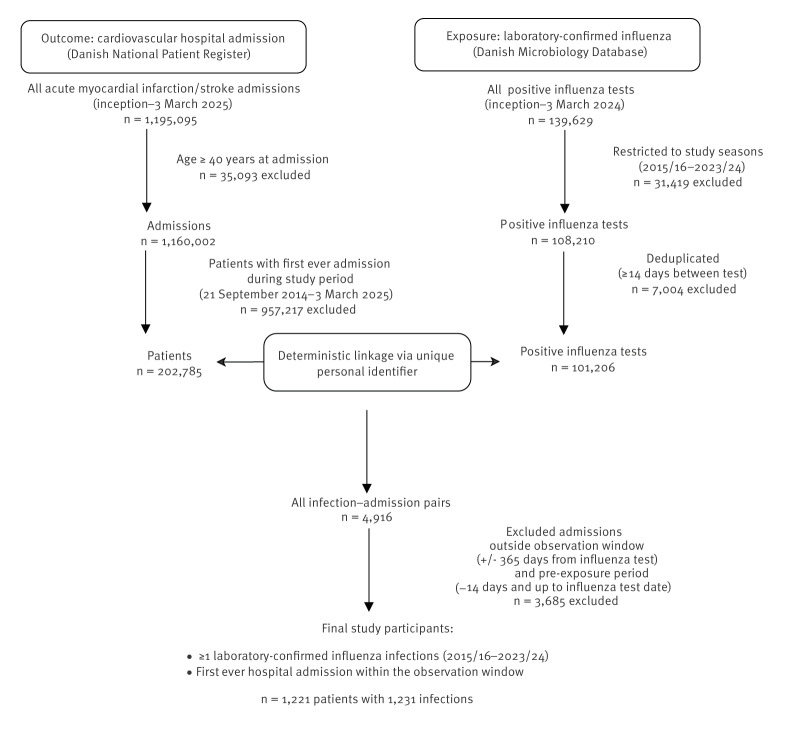
Data extraction flowchart of case selection to assess the effect of influenza vaccination on acute myocardial infarction and stroke risk following influenza infection, Denmark, 2014–2025 (n = 1,221)

From 21 September 2014 to 3 March 2025, we identified 202,785 first-ever hospital admissions for acute myocardial infarction or stroke in Denmark. Among these, 4,916 occurred in people with a PCR-confirmed influenza infection from the 2015/16 up to the 2023/24 influenza seasons. After excluding events outside the observation window or within the pre-exposure period, we accrued 1,221 individuals aged ≥ 40 years with a first-ever acute myocardial infarction or stroke admission and at least one temporally linked influenza infection. Figure 2 shows the data linkage process.

The study population included 660 males (54%) and 561 females (46%), with a median age of 75 years (interquartile range (IQR): 66–82). Among cardiovascular events, 792 (65%) were strokes, and 429 (35%) were acute myocardial infarctions. Median admission length was 2 days (IQR: 1–5). Ninety-two patients (7.5%) died within 30 days, and 282 (23%) at the end of follow-up (Table 1). Among strokes, ischaemic events predominated (644/792; 81%), most coded as unspecified cerebral infarction (568/792; 72%). Intracerebral haemorrhage accounted for 116 cases (15%) and subarachnoid haemorrhage for 32 (4%). Among acute myocardial infarctions, non–ST-elevation was most frequent (224/429; 52%), followed by 115 ST-elevation (27%), 79 unspecified (18%), and 11 type 2 myocardial infarction (3%). Full data on diagnostic subtypes are reported in Supplementary Tables S2 and S3.

**Table 1:** Study population characteristics: adults ≥ 40 years with first myocardial infarction or stroke admission and at least one confirmed influenza infection, Denmark, 21 September 2014–3 March 2025 (n = 1,221 patients)

Characteristic	Overall (n = 1,221)		Acute myocardial infarction (n = 429)		Stroke (n = 792)	
	n	%	n	%	n	%
Gender (ascertained administratively from the CPR register)						
Men	660	54	247	58	413	52
Women	561	46	182	42	379	48
Age at admission in years						
40–64	295	24	116	27	179	23
65–74	324	27	121	28	203	26
75–84	383	31	131	31	252	32
≥ 85	219	18	61	14	158	20
Length of hospital admission in days^a^						
0–2 days	691	57	266	62	425	54
3–7 days	311	25	116	27	195	25
8–30 days	178	15	42	10	136	17
31 days and more	41	3	5	1	36	4
Vital status 30 days after admission						
Deceased	92	8	26	6	66	8
Alive	1,129	92	403	94	726	92
Vital status at end of follow-up						
Deceased	282	23	96	22	186	23
Alive	939	77	333	78	606	77

CPR: Central Person Register.

^a^ Admission for specific diagnostic code, which might not reflect that patient’s entire hospital stay.

The 1,221 individuals contributed 1,231 influenza infection episodes, of which 610 (50%) were categorised as vaccinated, and 621 (50%) as unvaccinated. Most administered vaccinations were inactivated, corresponding to the Anatomical Therapeutic Chemical Classification System code J07BB02 (571/610; 94%). Of these, 42 were further classified as ‘high dose’, while the rest were standard dose. Influenza A accounted for 948 (77%) infections, with influenza B more common in 2017/18 (222/322; 69% for that season). Three consecutive seasons, namely 2016/17 to 2018/19, accounted for about half of the detections (584/1,231; 47%), while the 2020/21 season had one single infection. Influenza testing occurred outside the hospital admission in 1,214 episodes (99%), while 1% (17/1,231) were tested during admission. Vaccination took place most frequently between ISO weeks 39 and 45 (530 episodes; 87%) (Table 2). Supplementary Tables S4 and S5 provide data on vaccine and influenza virus types, respectively.

**Table 2:** Infection characteristics: adults ≥ 40 years with first myocardial infarction or stroke admission and at least one confirmed influenza infection, Denmark, 21 September 2014–3 March 2025 (n = 1,231 infections)

Characteristic	Overall (n = 1,231 infections)	
	n	%
Vaccination status		
Vaccinated^a^	610	50
Unvaccinated	621	50
Vaccination timing relative to influenza season (ISO weeks)		
Early: weeks 39–45	530	87
Mid-season: weeks 46–52	79	13
Later: week 53 to Week 9 of next calendar year	1	0
Influenza virus type		
Influenza A	948	77
Influenza B	283	23
Tested during cardiovascular hospital admission		
No	1,214	99
Yes	17	1

^a^ Influenza vaccine received ≥ 14 days before the positive test in the same influenza season.

### Primary analysis

There were 53 cardiovascular events during the risk period, amounting to a crude incidence of 2.28 per person-year, and 1,168 during the control period (0.53 per person-year).

In the conditional Poisson regression model, the unadjusted IRR was 4.2 (95% CI: 3.2–5.5). After calendar month adjustment, the IRR was 3.5 (95% CI: 2.6–4.7; p < 0.0001). By vaccination status, the adjusted IRRs were 4.7 (95% CI: 3.3–6.6) and 2.4 (95% CI: 1.5–3.8) for unvaccinated and vaccinated episodes, respectively. The IRR ratio was 0.51 (95% CI: 0.29–0.91; interaction p = 0.020).

Adjusted IRR was 4.7 (95% CI: 3.1–7.4) for acute myocardial infarction and 2.9 (95% CI: 2.0–4.2) for stroke. Vaccinated episodes had lower IRRs, but interaction terms were not statistically significant (p = 0.163 for myocardial infarction; p = 0.052 for stroke) (Table 3).

**Table 3:** Risk of cardiovascular hospitalisation in the 7 days after PCR-positive influenza test, overall and by influenza vaccination status, self-controlled case series study, Denmark, 21 September 2014–3 March 2025 (n = 1,221 patients)

Outcome	Exposure period	Vaccination status	Events (n)	Incidence per person-year	Adjusted IRR (95% CI)	Interaction p value^c^
Acute myocardial infarction and stroke (n = 1,221)	Control		1,168	0.53	1.0	Reference
	Risk^a^	Pooled	53	2.28	3.5 (2.6–4.7)	0.020
		Vaccinated^b^	18	1.57	2.4 (1.5–3.8)	
		Unvaccinated	35	2.97	4.7 (3.3–6.6)	
Acute myocardial infarction (n = 429)	Control		405	0.52	1.0	Reference
	Risk^a^	Pooled	24	2.96	4.7 (3.1–7.3)	0.163
		Vaccinated^b^	15	2.18	3.4 (1.7–6.7)	
		Unvaccinated	9	3.79	6.2 (3.6–11)	
Stroke (n = 792)	Control		763	0.53	1.0	Reference
	Risk^a^	Pooled	29	1.91	2.9 (2.0–4.2)	0.052
		Vaccinated^b^	9	1.23	1.8 (0.9–3.6)	
		Unvaccinated	20	2.56	3.9 (2.5–6.2)	

CI: confidence interval; IRR: incidence rate ratio.

^a^ Days 1–7 after influenza specimen collection date.

^b^ Influenza vaccine received ≥ 14 days before the positive test during the same influenza season.

^c^ Assessed by likelihood ratio test comparing models with and without interaction terms between vaccination status and risk period.

### Additional analyses

**Figure 3:**
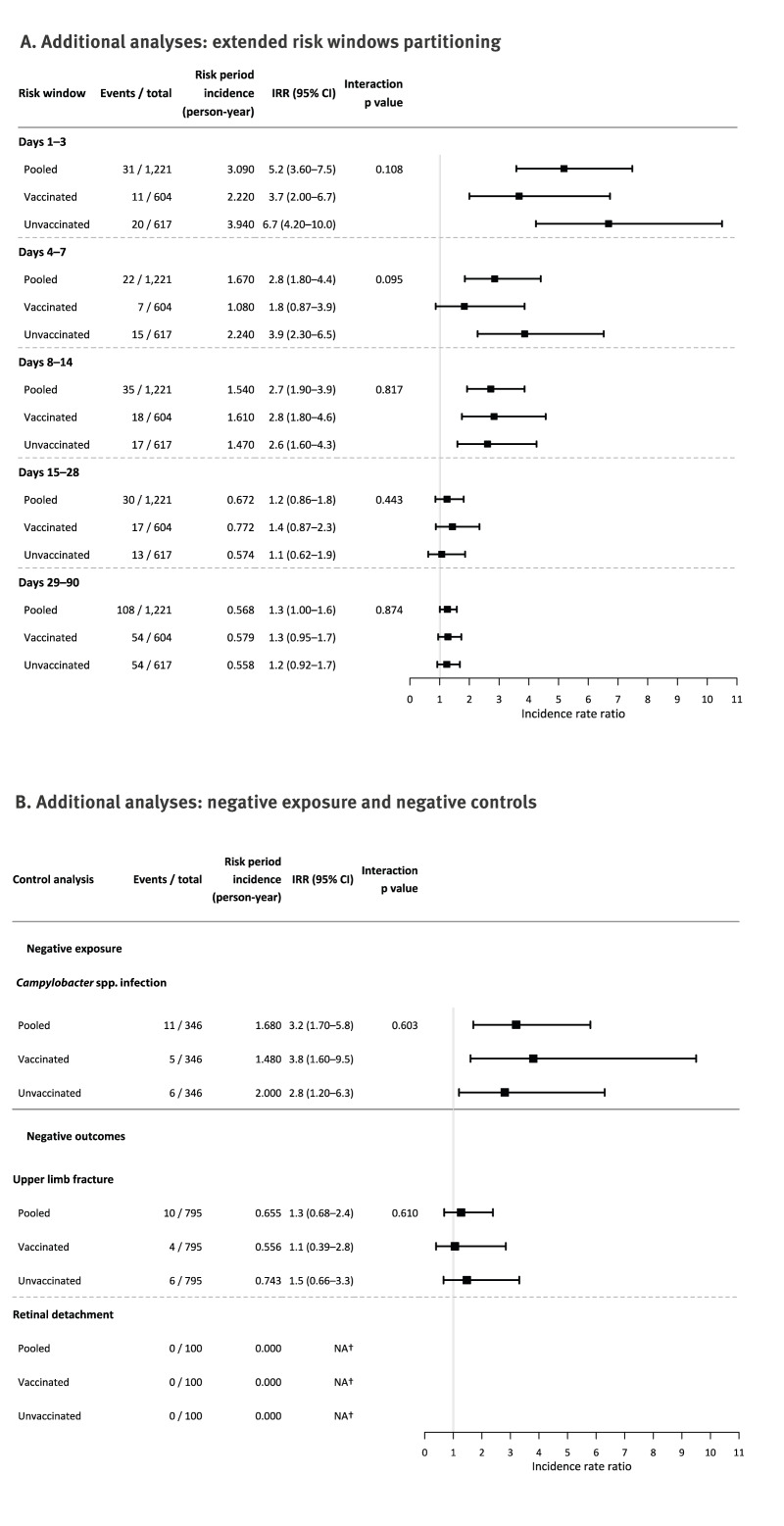
Forest plots of additional analyses: extended risk window partitioning, negative exposure and negative outcome analyses, effect of influenza vaccination on acute myocardial infarction and stroke risk following influenza infection, Denmark, 2014–2025 (n = 1,221)

CI: confidence interval; NA: not available; IRR: incidence rate ratio.

† Not available because there were 0 events in the days 1–7 risk window.

Partitioning the post-infection period showed a peak IRR of 5.2 on days 1–3 (95% CI: 3.6–7.5), followed by a return to the baseline by days 15–28 (IRR = 1.2; 95% CI: 0.9–1.8). Up to day 7, vaccinated episodes had lower adjusted IRRs than unvaccinated, although the interaction did not reach statistical significance (p = 0.108 for days 1–3; p = 0.095 for days 4–7). For later risk periods, there was no difference by vaccination status (Figure 3A).

In the negative control analyses, no retinal detachment events occurred during the risk period. For upper limb fracture, the pooled adjusted IRR was 1.3 (95% CI: 0.7–2.4). Repeating the primary analysis with *Campylobacter* spp. infection rather than influenza yielded a pooled adjusted IRR of 3.2 (95% CI: 1.7–5.8). No effect modification by influenza vaccination was observed (interaction p of approximately 0.60 in both analyses) (Figure 3B).

**Figure 4:**
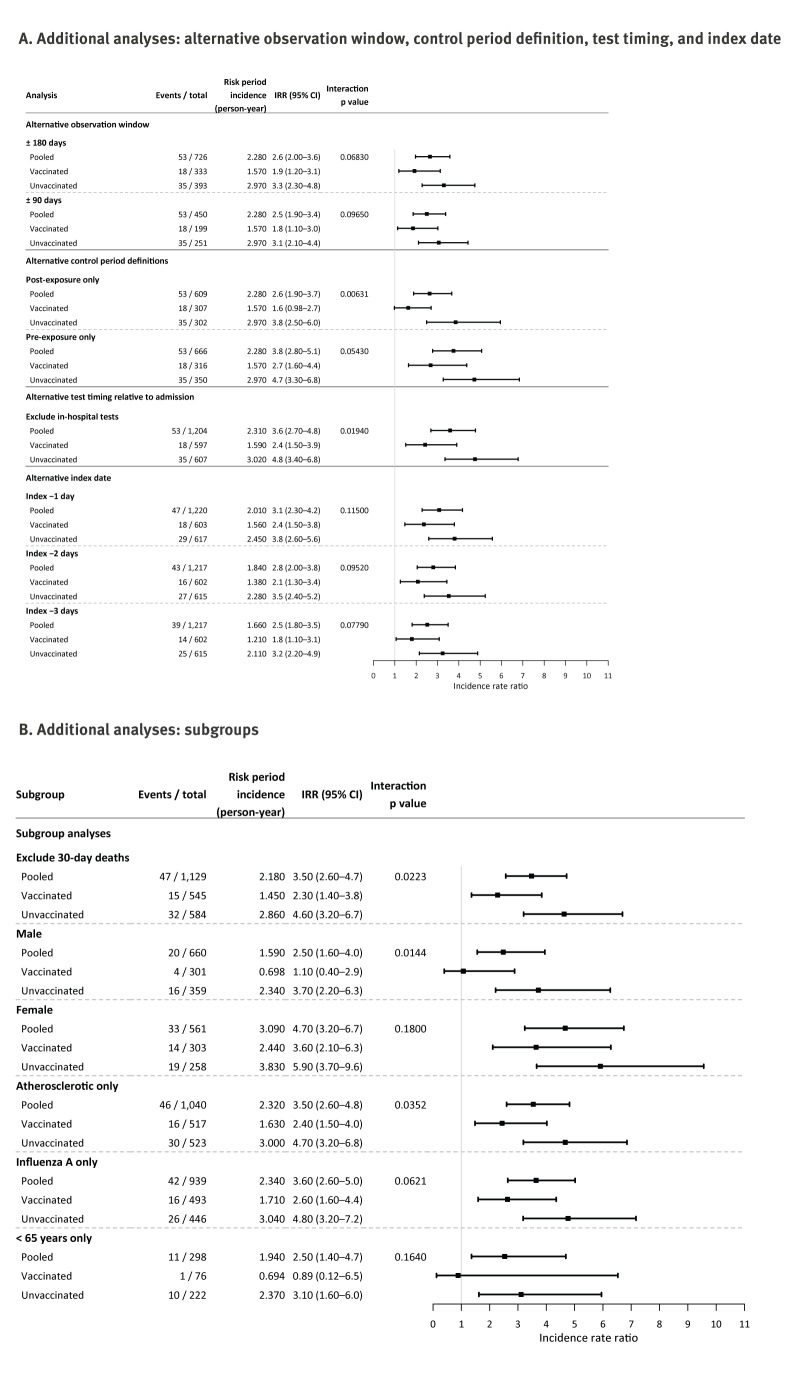
Forest plots of additional analyses: alternative time period and testing definitions and subgroups effect of influenza vaccination on acute myocardial infarction and stroke following influenza infection, Denmark, 2014–2025 (n = 1,221)

CI: confidence interval; IRR: incidence rate ratio.

Shortening the observation window to ± 180 days (pooled adjusted IRR = 2.6; 95% CI: 2.0–3.6) or ± 90 days (IRR = 2.5; 95% CI: 1.9–3.4) decreased estimates without changing their direction with respect to the primary analysis. Using only pre-exposure time as control resulted in a higher estimate (adjusted IRR = 3.8; 95% CI: 2.8–5.1) than only post-exposure time (IRR = 2.6; 95% CI: 1.9–3.7). In both cases, vaccination remained associated with a lower excess risk. Excluding patients with in-hospital testing produced no change (pooled IRR = 3.6; 95% CI: 2.7–4.8). Shifting the index date backward to approximate true symptom onset decreased pooled estimates (index −1 day: IRR = 3.1; 95% CI: 2.3–4.2; index −2 days: IRR = 2.8; 95% CI: 2.0–3.8; index −3 days: IRR = 2.5; 95% CI: 1.8–3.5), preserving both the temporal pattern and difference by vaccination (Figure 4A).

Adjusted IRRs were 2.5 (95% CI: 1.6–4.0) in males and 4.7 (95% CI: 3.2–6.7) in females, lower for vaccinated episodes for both. Point estimates remained unaltered from the overall model after excluding 30-day deaths (pooled IRR = 3.5; 95% CI: 2.6–4.7) and when restricted to influenza A (pooled IRR = 3.6; 95% CI: 2.6–5.0). Among persons ≤ 65 years, the rate ratio was lower than in the main model (pooled IRR = 2.5; 95% CI: 1.4–4.7). Restricting to atherosclerotic events yielded a pooled IRR of 3.5 (95% CI: 2.6–4.8), remaining lower in vaccinated episodes (Figure 4B). Age-adjustment approaches altered IRRs at the first decimal place: we provide these details in Supplementary Table S1.

## Discussion

In this self-controlled case series study in Denmark spanning 2014 to 2025, PCR-confirmed influenza was followed by a sharp, transient rise in the first-ever hospitalisations for acute myocardial infarction and stroke. Risk concentrated in the first week, peaking within 3 days, and declined back to baseline by 2 weeks. Prior influenza vaccination was associated with a significantly lower excess risk.

This temporal profile aligns with studied mechanisms. Influenza infection has been shown to precipitate atherogenesis and has been epidemiologically linked to acute myocardial infarction and stroke in adults 40 years and older [22,23]. Vaccination can plausibly mitigate these effects by priming adaptive immunity and reducing viral replication, thereby dampening systemic inflammatory peaks. To our knowledge, this is the first study to show statistically significant attenuation of post-influenza cardiovascular risk by vaccination. A Canadian study observed similar results but possibly lacked statistical power to confirm them [5]. By including both acute myocardial infarction and stroke across a larger nationwide dataset, we demonstrate statistically significant effect modification.

Our risk estimates for myocardial infarction (ca 4.7) and stroke (ca 2.9) align with a recent meta-analysis [24]. Among studies of comparable sample size, only a veteran cohort from the United States reported higher estimates (7.0 for acute myocardial infarction; 4.0 for ischaemic stroke) [25], but their lack of seasonality adjustment might have led to inflated estimates. Their study excluded in-hospital tests; a similar exclusion did not affect our results because such tests were rare.

A meta-analysis of randomised controlled trials reported a 45% reduction in the risk of cardiovascular death or hospitalisation (risk ratio = 0.55; 95% CI: 0.41–0.75) among influenza-vaccinated vs unvaccinated individuals with recent acute coronary syndrome [10]. Previous self-controlled case series studies focusing on vaccination irrespective of infection status found a modest protective effect against acute myocardial infarction lasting up to 28 days after vaccination [11-13]. These findings do not clarify if protection arose from preventing infection, mitigating its consequences, or both. Our study addresses this ambiguity by restricting the study population to individuals with a confirmed influenza infection.

Smeeth et al. [11] explored the cardiovascular effects of respiratory infection and vaccination separately, reporting a fourfold increase in cardiovascular risk following clinically diagnosed respiratory tract infection, and a small protective effect of influenza vaccination, which they attributed in part to its timing during periods of relatively good health. To minimise such bias, we did not treat vaccination as a time-varying exposure in our study. Instead, we classified each influenza episode as vaccinated or unvaccinated based on vaccine receipt before infection within the same season.

Our study has several strengths. The self-controlled case-series design adjusts for all time-invariant individual confounders, including comorbidities, genetic predisposition and socioeconomic status [4]. We defined influenza exposure by PCR positivity and ascertained vaccination status and clinical outcomes from complete national records. We prespecified a pre-exposure exclusion period to reduce reverse causality and we ran multiple additional analyses. Overall, these confirmed robustness to alternative control period definitions, observation windows and exclusion of in-hospital testing. To test for unmeasured confounding, we used negative-control outcomes for which we hypothesised no causal link to influenza.

We acknowledge several limitations. As symptom onset dates were unavailable, we used specimen date as exposure anchor, which could potentially cause an underestimation of the excess risk. Additional analyses with shifted index dates preserved the risk pattern.

Unexpectedly, infection with *Campylobacter* spp., a food-borne, non-respiratory bacterial pathogen, was associated with a threefold increase in acute cardiovascular events. One explanation may be that any infection severe enough to warrant microbiological testing can result in proinflammatory events. Alternatively, unmeasured respiratory co-infections might have determined the risk increase, at least in part. Meta-analyses have shown that respiratory pathogens other than influenza can trigger acute cardiovascular events [24]. Research should clarify whether this is true for non-respiratory pathogens as well. Importantly, the absence of effect modification by influenza vaccination in our *Campylobacter*-negative exposure analysis reinforces that the observed risk attenuation after influenza infection is exposure-specific. *Campylobacter*-induced myocarditis, which might mimic acute myocardial infarction, has been reported [26]. This clinical presentation alone is unlikely to account for our findings, considering its rarity and the high diagnostic specificity for acute myocardial infarction diagnosis in Denmark.

Severe health events, such as the outcomes we studied, influence survival; therefore, censoring was not event-independent in our study. We addressed this potential source of bias through ad hoc analyses that considered pre-only or post-only control periods and excluded 30-day deaths. We ascertained outcomes using hospital administrative coding, which, in principle, might introduce misclassification. This is mitigated by the extremely high coding specificity for acute myocardial infarction in Denmark, with stroke having an inferior but still acceptable specificity. We believe such misclassification is non-differential with respect to the time periods and would dilute associations. A high proportion of acute myocardial infarction and stroke was coded as ‘unspecified’, preventing us from studying outcomes in more detail.

Our study design does not account for heterogeneity in influenza vaccine effectiveness. We determined vaccination status based on receipt of seasonal vaccine, but vaccine effectiveness varies annually depending on viral evolution and vaccine match. This variability alone could alter the observed effect modification if, for example, vaccinated and unvaccinated individuals in seasons with poor vaccine effectiveness experienced equally elevated cardiovascular risks post-infection. Our analysis assumes a consistent biological effect of vaccination in reducing influenza-associated cardiovascular triggers. To understand if this assumption is realistic, future studies should integrate vaccine effectiveness data to refine risk estimates.

The study population consisted of Danish residents aged 40 years and older with laboratory-confirmed influenza, and milder infections are probably underrepresented. Results may not apply directly to populations or settings with different influenza epidemiology, healthcare systems or vaccination strategies.

One previous study showed a stronger cardioprotective effect of influenza vaccination among people vaccinated early in the season, with no gender-specific differences [12]. Within our subgroups, females had a higher relative incidence of cardiovascular events following influenza than males, with adjusted IRRs of 4.7 and 2.5, respectively. In contrast, vaccination appeared more protective in males. This pattern should be interpreted very cautiously, as only four vaccinated males experienced a qualifying event. The observed difference is, therefore, more likely to be attributable to random variation than to a true biological effect.

We could not assess whether vaccination timing influenced effect modification, as 87% of vaccinated episodes occurred early in the season, leaving insufficient contrast for a meaningful analysis. Future studies with larger stratum-specific samples could clarify these observations. Similarly, our study could not address differences by vaccine type. Two recent randomised controlled trials found greater protection against hospitalisation for cardiorespiratory disease with high-dose influenza vaccines than with standard-dose vaccines [27]. We cannot replicate these findings for the interaction effect on cardiovascular hospitalisations, as only 42 high-dose vaccinations were recorded in the study. Studying type-specific secondary prevention benefits of influenza vaccines might represent a promising new research line.

We cannot exclude residual time-varying confounding from co-circulating pathogens, weather fluctuations or temporary health-system strain. Such influences are not adequately captured by calendar month adjustment. However, our negative control outcome analyses were reassuring. We found no retinal detachment event associated with influenza exposure, and upper limb fractures were not associated with increased risk.

## Conclusions

Our findings add to the evidence that influenza vaccination confers cardiovascular protection. In this study, prior vaccination halved the excess risk of acute myocardial infarction or stroke following breakthrough influenza infection. These results strengthen the case for prioritising influenza vaccination in high-risk groups. Highlighting the dual protection offered by vaccination, against both infection and its cardiovascular complications, could have a substantial public health impact. Factoring this into economic and burden analysis might improve the cost-effectiveness profile of vaccination programmes.

## Data Availability

The study protocol (R scripts) for the self-controlled case series analysis is available upon request from the corresponding author. Deidentified participant-level data are available for access to members of the scientific and medical community for non-commercial use only. Applications for data access should be submitted to Forskerservice at The Danish Health Data Authority and will be reviewed based on relevance and scientific merit. Data are available now, with no defined end date. For information about applying, see the Forskerservice website (https://sundhedsdatastyrelsen.dk/da/forskerservice).

## Note

At the time of article submission, Roberto Croci was a fellow of the ECDC Fellowship Programme, supported financially by the European Centre for Disease Prevention and Control. The views and opinions expressed herein do not state or reflect those of ECDC. ECDC is not responsible for the data and information collation and analysis and cannot be held liable for conclusions or opinions drawn.

## Supplementary Data

534000 Supplement
