# Supplementary material for: Influenza vaccination attenuates acute myocardial infarction and stroke risk following influenza infection: a register-based, self-controlled case series study, Denmark, 2014 to 2025
Source: Euro Surveill. 2026 Apr 2;31(13):2500706. doi: 10.2807/1560-7917.ES.2026.31.13.2500706 (PMC13074456; doi:10.2807/1560-7917.ES.2026.31.13.2500706)

Supplementary Materials

This supplementary material is hosted by *Eurosurveillance* as supporting information alongside the article "Influenza vaccination attenuates acute myocardial infarction and stroke risk following influenza infection: a register-based, self-controlled case series study, Denmark, 2014-2025", on behalf of the authors, who remain responsible for the accuracy and appropriateness of the content. The same standards for ethics, copyright, attributions and permissions as for the article apply. Supplements are not edited by *Eurosurveillance* and the journal is not responsible for the maintenance of any links or email addresses provided therein.

Table of contents

Statistical model 3

Supplementary tables 4

Table S1. Age adjustments. 4

Table S2. Stroke subtypes (n = 792) by Danish National Patient Register (DNPR) diagnostic codes and corresponding International Classification of Diseases-10 (ICD-10) codes. 5

Table S3. Acute myocardial infarction subtypes (n = 429), by DNPR diagnostic codes and corresponding ICD-10 codes. 7

Table S4. Vaccine type received by influenza season and vaccine type (n = 610 vaccinated influenza episodes). 8

Table S5. Number and proportion of PCR-confirmed influenza samples by influenza season and virus subtype. 9

Table S6. Event sequences (n = 1,221 included patients). 10

Table S7.1. Comparison of patients' baseline characteristics at the start of study follow-by vaccination status (n = 1,221 included patients). 11

Table S7.2. ICD-10 codes used to define patient comorbidities. 12

Table S8. Investigation of potential confounding by participants' characteristics. 13

Supplementary figures 14

Figure S1. Distribution of AMI and stroke hospitalisations relative to influenza specimen date. 14

Statistical model

For the primary analysis, we employed a self-controlled case series design, fitting individual fixed-effect Poisson models with the `fepois` function from the fixest R package (v 0.12.1)[1] to estimate the relative incidence of acute cardiovascular events (AMI/stroke) following PCR-confirmed influenza infection.

For each PCR-confirmed influenza episode, starting at index date = day 0, the main risk interval was defined as 1–7 days post-sample date, with control period incidence derived from person-time outside these intervals (365 days pre-infection excluding a 14-day pre-exposure period and 8–365 days post-infection).

Primary model. The base model adjusted for calendar month as a 12-level categorical covariate, and was specified as:

$$\log \lambda_{it} = \alpha_i + \log(person\_days_{it}) + \beta_1 \, I_{1-7}(t) + f_{calendar}(t)$$

Where: λ_{it} is the expected Poisson rate of a cardiovascular event at time t for individual I, α_i, are individual fixed effects, $I_{1-7}(t)$ indicates the post-infection risk interval (days 1-7), and $f_{calendar}(t)$ denotes month dummies.

To assess effect modification by influenza vaccination, Vaccination status V was defined at the episode level: vaccinated if a seasonal influenza vaccine had been administered ≥14 days before the index date within the same season (ISO week 39 to week 9 of next calendar year); otherwise unvaccinated. Effect modification was assessed via an interaction between the risk indicator and V:

$$\log \lambda_{it} = \alpha_i + \log(person\_days_{it}) + \beta_1 \, I_{1-7}(t) + \beta_2 [I_{1-7}(t) \times V] + f_{calendar}(t)$$

where V indicates vaccination status.

In supplementary analyses, age was updated monthly and included as either natural cubic splines at 10th, 50th, and 90th quantiles, or, alternatively with a linear or a quadratic term.

Models were fitted via conditional Poisson regression with log(person-days) as an offset, with individual fixed effects. Relative incidence ratios (IRRs) and 95% confidence intervals were derived from exponentiated coefficients, with significance assessed via Wald tests (α=0.05). Interaction significance was assessed by likelihood-ratio test comparing models with and without the $[I_{1-7}(t) \times V]$ term.

[1] Package "fixest" manual: https://cran.r-project.org/web/packages/fixest/fixest.pdf

Supplementary tables

Table S1. Age adjustments.

Adjustment set	Pooled adjusted IRR (95% CI)	Vaccinated adjusted IRR (95% CI)	Unvaccinated adjusted IRR (95% CI)	AIC[a]	BIC[b]
None (model not adjusted for calendar month)	4.20 (3.19–5.53)	2.82 (1.77–4.50)	5.60 (3.98–7.88)	12,745.71	22,922.43
**Models with calendar month adjustment and additional age adjustment**					
Age updated monthly, linear term	3.43 (2.57–4.57)	2.31 (1.44–3.72)	4.57 (3.21– 6.51)	12,710.45	22,987.03
Age updated monthly, quadratic term	3.43 (2.57–4.57)	2.31 (1.44–3.72)	4.57 (3.21–6.51)	12,708.85	22,993.74
Age updated monthly, splines with 3 degrees of freedom	3.43 (2.57–4.57)	2.31 (1.44–3.71)	4.57 (3.21–6.51)	12,707.64	23,009.18
Age updated monthly, splines with 4 degrees of freedom	3.43 (2.58–4.58)	2.31 (1.44–3.71)	4.58 (3.22–6.52)	12,706.06	23,015.92
Age updated monthly, splines with 5 degrees of freedom	3.43 (2.57–4.58)	2.30 (1.43–3.71)	4.59 (3.22–6.53)	12,706.01	23,024.18

a AIC: Akaike's Information Criterion.

b BIC: Bayes Information Criterion.

Table S2. Stroke subtypes (n = 792) by Danish National Patient Register (DNPR) diagnostic codes and corresponding International Classification of Diseases-10 (ICD-10) codes.

DNPR diagnostic code	Description	ICD-10 code	Description	n	(%)
DI606B	Subaraknoidalblødning fra arteria cerebri anterior	I60.6	Nontraumatic subarachnoid haemorrhage from other intracranial arteries	1	0.13
DI601	Subaraknoidalblødning fra arteria cerebri media	I60.1	Nontraumatic subarachnoid haemorrhage from middle cerebral artery	1	0.13
DI602	Subaraknoidalblødning fra arteria communicans anterior	I60.2	Nontraumatic subarachnoid haemorrhage from anterior communicating artery	3	0.38
DI607	Subaraknoidalblødning fra intrakraniel arterie UNS	I60.7	Nontraumatic subarachnoid haemorrhage from unspecified intracranial artery	3	0.38
DI608	Anden form for subaraknoidalblødning	I60.8	Other nontraumatic subarachnoid haemorrhage	2	0.25
DI609	Subaraknoidalblødning UNS	I60.9	Nontraumatic subarachnoid haemorrhage, unspecified	22	2.78
DI610	Subkortikal blødning i hjernehemisfære	I61.0	Nontraumatic intracerebral haemorrhage in hemisphere, subcortical	10	1.27
DI610A	Dybtliggende blødning i hjernehemisfære				
DI611A	Blødning i hjernens overflade	I61.1	Nontraumatic intracerebral haemorrhage in hemisphere, cortical	1	0.13
DI612	Intracerebral blødning i hjernehemisfære UNS	I61.2	Nontraumatic intracerebral haemorrhage in hemisphere, unspecified	25	3.16
DI613	Blødning i hjernestammen	I61.3	Nontraumatic intracerebral haemorrhage in brain stem	2	0.25
DI614	Blødning i lillehjernen	I61.4	Nontraumatic intracerebral haemorrhage in cerebellum	2	0.25
DI615	Blødning i hjerneventrikel	I61.5	Nontraumatic intracerebral haemorrhage, intraventricular	4	0.51

DNPR diagnostic code	Description	ICD-10 code	Description	n	(%)
DI618	Anden form for hjerneblødning	I61.8	Other nontraumatic intracerebral haemorrhage	5	0.63
DI619	Hjerneblødning UNS	I61.9	Nontraumatic intracerebral haemorrhage, unspecified	67	8.46
DI630	Hjerneinfarkt forårsaget af trombose i præcerebral arterie	I63.0	Cerebral infarction due to thrombosis of precerebral arteries	3	0.38
DI631	Hjerneinfarkt forårsaget af emboli i præcerebral arterie	I63.1	Cerebral infarction due to embolism of precerebral arteries	1	0.13
DI632	Hjerneinfarkt forårsaget af tillukning eller stenose i præcerebral arterie UNS	I63.2	Cerebral infarction due to unspecified occlusion or stenosis of precerebral arteries	15	1.89
DI633	Hjerneinfarkt forårsaget af trombose i cerebral arterie	I63.3	Cerebral infarction due to thrombosis of cerebral arteries	17	2.15
DI634	Hjerneinfarkt forårsaget af emboli i cerebral arterie	I63.4	Cerebral infarction due to embolism of cerebral arteries	14	1.77
DI635	Hjerneinfarkt forårsaget af tillukning eller stenose i cerebral arterie UNS	I63.5	Cerebral infarction due to unspecified occlusion or stenosis of cerebral arteries	22	2.78
DI638	Anden form for hjerneinfarkt	I63.8	Other cerebral infarction	4	0.51
DI639	Hjerneinfarkt UNS	I63.9	Cerebral infarction, unspecified	568	71.72
Total				792	100.00

DNPR codes descriptors extracted from: https://www.medinfo.dk/sks/brows.php.

ICD-10 corresponding codes and descriptors extracted from: https://www.icd10data.com/search?s=I63.9

Table S3. Acute myocardial infarction subtypes (n = 429), by DNPR diagnostic codes and corresponding ICD-10 codes.

DNPR diagnostic code	Description	ICD-10 code	Description	n	(%)
DI210	Anteriort akut myokardieinfarkt med Q-taksudvikling	I21.0	ST elevation (STEMI) myocardial infarction of anterior wall	18	4.20
DI210B	Anteriort ST-elevations akut myokardieinfarkt med Q-taksudvikling				
DI213A	ST-elevations akut myokardieinfarkt, anteriort				
DI211	Inferiort eller posteriort akut myokardieinfarkt med Q-taksudvikling	I21.1	ST elevation (STEMI) myocardial infarction of inferior wall	26	6.06
DI211B	Inferiort eller posteriort ST-elevations akut myokardieinfarkt med Q-taksudvikling				
DI213B	ST-elevations akut myokardieinfarkt, inferiort/posteriort				
DI213	ST-elevations akut myokardieinfarkt	I21.3	ST elevation (STEMI) myocardial infarction of unspecified site	71	16.55
DI210A	Anteriort non-ST-elevations akut myokardieinfarkt med Q-taksudvikling	I21.4	Non-ST elevation (NSTEMI) myocardial infarction	224	52.21
DI211A	Inferiort eller posteriort non-ST-elevations akut myokardieinfarkt med Q-taksudvikling				
DI214	Non-ST-elevations akut myokardieinfarkt				
DI219	Akut myokardieinfarkt UNS	I21.9	Acute myocardial infarction, unspecified	79	18.41
DI219A	Type 2 myokardieinfarkt	I21.A1	Myocardial infarction type 2	11	2.56
Total				429	100.00

Danish codes descriptors from: https://www.medinfo.dk/sks/brows.php. ICD-10 corresponding codes and descriptors extracted from: https://www.icd10data.com/search?s=I21.For acute myocardial infarction, the Danish coding system still uses ICD8/9 some nomenclature ("Q waves"), which we reconciled with ICD-10 codes for comparability.

Table S4. Vaccine type received by influenza season and vaccine type (n = 610 vaccinated influenza episodes).

Influenza season	Inactivated (split or subunit)[a]		Influenza vaccine, unspecified		Total (n)	% of all vaccinations
	n	(%)	n	(%)		
2015/2016	22	100	0	0	22	4
2016/2017	47	100	0	0	47	8
2017/2018	137	100	0	0	137	22
2018/2019	79	100	0	0	79	13
2019/2020	38	100	0	0	38	6
2020/2021	1	100	0	0	1	0
2021/2022	81	70	34	30	115	19
2022/2023	86	95	5	5	91	15
2023/2024	80	100	0	0	80	13
Total	571[b]	94	39	6	610	100

a Anatomical Therapeutic Chemical Classification System code J07BB02.

b including 42 vaccines classified as "high dose".

Table S5. Number and proportion of PCR-confirmed influenza samples by influenza season and virus subtype.

Influenza season	Influenza A		Influenza B		Total (n)	% of all samples
	n	(%)	n	(%)		
2015/2016	38	54.3	32	45.7	70	5.7
2016/2017	100	97.1	3	2.9	103	8.4
2017/2018	100	31.1	222	68.9	322	26.2
2018/2019	157	98.7	2	1.3	159	12.9
2019/2020	92	98.9	1	1.1	93	7.6
2020/2021	1	100.0	0	0.0	1	0.1
2021/2022	156	99.4	1	0.6	157	12.8
2022/2023	129	86.6	20	13.4	149	12.1
2023/2024	175	98.9	2	1.1	177	14.4
Total	948	77.0	283	23.0	1,231	100.0

Table S6. Event sequences (n = 1,221 included patients).

Sequence	Description	n	%
1	Cardiovascular hospitalisation followed by influenza infection	315	25.8
2	Influenza vaccination, followed by influenza infection, followed by cardiovascular hospitalisation	306	25.1
3	Influenza infection followed by cardiovascular hospitalisation	302	24.7
4	Cardiovascular hospitalisation, followed by influenza vaccination, followed by influenza infection	181	14.8
5	Influenza vaccination, followed by cardiovascular hospitalisation, followed by influenza infection	117	9.6

Table S7.1. Comparison of patients' baseline characteristics at the start of study follow-by vaccination status (n = 1,221 included patients).

Characteristic	Vaccinated (n = 604)	Unvaccinated (n = 617)	p-value
Age	76 (70-81)	70 (59-79)	<0.0001[a]
Males	301 (49.8%)	359 (58.2%)	0.0034[b]
Type 2 diabetes mellitus	98 (16.2%)	88 (14.3%)	0.34[b]
Overweight or obesity	57 (9.4%)	43 (7.0%)	0.12[b]
Cancer anamnesis	23 (3.8%)	7 (1.1%)	0.0025[b]
Chronic kidney disease or dialysis	46 (7.6%)	48 (7.8%)	0.91[b]
Chronic liver disease	10 (1.7%)	7 (1.1%)	0.44[b]
Chronic respiratory diseases	164 (27.2%)	81 (13.1%)	<0.0001[b]
Selected immunocompromising conditions	19 (3.1%)	12 (1.9%)	0.18 [b]

Data are median and interquartile range for age and n (%) for all other variables.

Comorbidities are extracted from the Danish National Patient Register and measured at the point in time where patients enter the study, that is, 365 days before their influenza specimen collection date.

a Two-sided Wilcoxon rank-sum test with continuity correction.

b Two-sided Chi square test.

Table S7.2. ICD-10 codes used to define patient comorbidities.

Diagnosis	ICD-10 code and description
Diabetes mellitus type 2	E11, "Type 2 diabetes mellitus"
Overweight or obesity	E66, "Overweight and obesity"
Cancer anamnesis	Z85, "Personal history of malignant neoplasm"
Chronic kidney disease or dialysis	N18, "Chronic kidney disease"; N19, "Unspecified kidney failure"; Z99, "dependence on renal dialysis"
Chronic liver disease	K70, "Alcoholic liver disease"; K73, "Chronic hepatitis, not elsewhere classified"; K75, "Other inflammatory liver diseases"; B18, "Chronic viral hepatitis"
Chronic respiratory disorders	J40, "Bronchitis, not specified as acute or chronic"; J41, "Simple and mucopurulent chronic bronchitis"; J42, "Unspecified chronic bronchitis"; J43, "Emphysema"; J44, "Other chronic obstructive pulmonary disease"; J45, "Asthma"; J47, "Bronchiectasis"; J4A, "Chronic lung allograft dysfunction"
Selected immunocompromising conditions	Z94, "Organ or tissue replaced by transplant"; Z51.0, "Encounter for radiotherapy"; Z51.1, "Encounter for antineoplastic chemotherapy"; D80–D84, "Immunodeficiency"; Z90.8, "Acquired absence of other organs (including spleen)"

Table S8. Investigation of potential confounding by participants' characteristics.

We explored potential confounding by the indicator variables listed in Table 7.1 by adding them one by one to the main model through an additional interaction term in the linear predictor, as shown below, where we have used X to denote the potential confounder being investigated taking the value 1 for individuals with the characteristic at hand and 0 otherwise. The model equation is as follows:

$$\log \lambda_{it} = \alpha_i + \log(person\_days_{it}) + \beta_1 \ I_{1-7}(t) + \beta_2[I_{1-7}(t) \ \times V] + \beta_3[\ X \times V] + \ f_{calendar}(t)$$

	Effect of vaccination, beta2 (95% CI)	Exponentiated coefficient (95% CI)
Main model	-0.672 (-1.252 to-0.092)	0.511 (0.286 to 0.912)
Potential confounder:		
Age[a]	-0.864 (-1.476 to -0.253)	0.421 (0.229 to 0.776)
Male gender	-0.731 (-1.307 to -0.155)	0.481 (0.271 to 0.856)
Type 2 diabetes mellitus	-0.672 (-1.253 to -0.092)	0.511 (0.286 to 0.912)
Overweight or obesity	-0.662 (-1.244 to -0.079)	0.516 (0.288 to 0.924)
Cancer anamnesis	-0.673 (-1.25 to -0.096)	0.510 (0.287 to 0.909)
Chronic kidney disease or dialysis	-0.672 (-1.252 to -0.092)	0.511 (0.286 to 0.912)
Chronic liver disease	-0.671 (-1.251 to -0.091)	0.511 (0.286 to 0.913)
Chronic respiratory diseases	-0.662 (-1.246 to -0.078)	0.516 (0.288 to 0.925)
Selected immunocompromising conditions	-0.659 (-1.239 to -0.079)	0.517 (0.289 to 0.924)

a Dichotomised at the pooled median (0 if age is below 75, 1 if it is 75 or above).

Interpretation: as the beta2 estimates shown in the table are very similar, we can conclude that there is no or little confounding by the variables listed.

Supplementary figures

Figure S1. Distribution of AMI and stroke hospitalisations relative to influenza specimen date.

Week 1, in blue, corresponds to the predefined 7-day risk period. All other weeks are shown in grey and represent the control period. The pre-exposure window (days –14 to 0; index date = date of positive influenza test) does not contribute events, and therefore no bar is shown. The dashed line denotes the median number of weekly hospitalisations during the control period. Weeks are numbered according to ISO week conventions, with 53 weeks per calendar year in the symmetrical observation window before and after the index date.

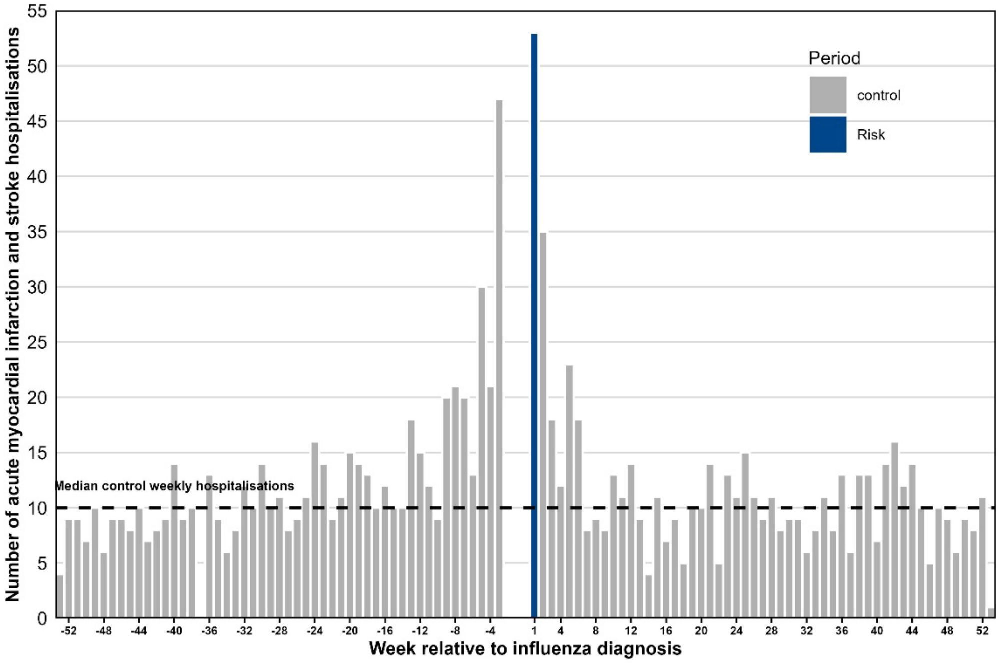

Supplement: Supplement [file 25-00706_CROCI_Supplement.pdf]
